# Menarche education and emotional preparedness: A cross-sectional survey study of Chinese adolescent girls

**DOI:** 10.1016/j.heliyon.2025.e42904

**Published:** 2025-02-21

**Authors:** Yifei Gao, Jue Wang, Mulan He, Ruhe Jiang, Yan Du, Qi Chen

**Affiliations:** a Obstetrics and Gynaecology Hospital, Fudan University, Shanghai, 200011, China; b Department of Obstetrics and Gynaecology, The University of Auckland, Auckland, New Zealand

**Keywords:** Adolescence, Puberty education, Menarche, Knowledge on menarche, Emotional impacts, low- and middle-income countries

## Abstract

Adolescent girls undergo a transformative period of both physical and emotional changes. Menarche, the first onset of the menstrual cycle, is one of the physical changes, and this could be a challenging time for the girls. For many, menarche remains a difficult experience in both developed and developing countries due to limited understanding and awareness of this process. An inadequate explanation of menarche by the family or society can lead to positive emotions during this transition. Unfortunately, limited availability of puberty education persists, particularly in low- and middle-income countries, including China. In this cross-sectional paper-based survey study with a moderate sample size, we evaluated Chinese adolescent girls' knowledge and emotional responses to menarche. The study included 2,032 teenage girls aged 10 to 17 from primary and middle schools in Shanghai and Yunnan Province, China. The mean age at menarche was 12.7 years. 36 % of the participants did not receive any information about menarche before their first menstrual cycle. Mothers, rather than schools, were the primary source of knowledge about menarche and also the main individuals girls sought advice from. The school provided limited knowledge on menstruation, leaving many girls without the necessary knowledge. Additionally, 57 % of adolescent girls reported experiencing varying degrees of negative emotions regarding menarche. Given that the average age of pubertal onset is now below ten years, our paper-based survey highlights the growing importance of puberty education, including stating earlier that it needs to be a high priority in managing adolescent girls’ health, at least at the community level in low- and middle-income countries.

## 1 Introduction

Adolescence is the developmental period between childhood and adulthood with physical and psychological changes. This transformative period naturally triggers anxiety due to hormonal and biological changes, leading to issues such as sexual curiosity and other risky behaviors unique to adolescents. One notable physical development during adolescence is menarche, the first onset of the menstrual cycle. Unlike other normal bodily processes, menarche is one of the most pivotal milestones in a female's life. It is also associated with the development of secondary sexual characteristics and links to the transformative period from a girl to a woman [1,2], as the reproductive organs become functionally active.

It is now considered that menarche is not merely a physiological change and can be viewed as the main event of adolescence and emblematic of the transformative period to adulthood. However, several factors, such as environmental, nutritional, and geographical factors, could affect the age of menarche [3]. Consequently, for many girls, menarche can still be a challenging time in many developed and developing countries, as understanding and awareness of this process may be limited [4].

Girls are likely to have a positive experience of menarche if menarche is adequately explained by the family or society [5]. However, several vital factors could impact the girls' understanding of menarche. According to some traditional cultures in developing countries, mothers (and sometimes fathers) may feel uncomfortable talking to their daughters about menstruation due to its association with sexuality [6]. Due to the personal nature of menarche, in addition to the lack of public puberty education programs, they may be unsure who should ask and how to ask for advice (reviewed in Ref. [7]). Social media may also lead girls to feel that menarche is dirty, shameful, uncomfortable, or unhealthy [8,9]. Therefore, there is a need to approach a better understanding of menarche to provide better adolescent healthcare, given its substantial public health importance, especially for teenage girls who need it.

The industrialization and Westernization that started in the 1980s in China led to significant changes in Chinese traditional cultures and lifestyles. Studies have recently shown a decline in the mean age at menarche in Chinese girls, from 14.3 years to 12.3 years between 1976 and 2014 [10,11]. However, due to the lack of puberty education programs in China, adolescent girls may still need more knowledge and awareness about menarche, including medical conditions related to abnormal uterine bleeding.

A recent review study reported that adolescent girls in low-middle-income countries need to be more informed and prepared for menarche [7]. In response to this need, our group initially started a puberty education program focusing on the puberty changes of reproductive organs and medical knowledge in this perspective in 2013 in Shanghai. Further, we promoted it to Xiangyun County, Yunan province (located southwest of China and bordering Myanmar, Laos, and Vietnam) in 2019. This program is now well-received by adolescent girls.

To improve our current puberty education program, we performed a quantitative survey using menarche as a marker of normal female reproductive health and well-being, aiming to evaluate (1) What kind of knowledge about menarche did Chinese adolescent girls have? (2) Where the information about menarche can be obtained from for adolescent girls. Improving the knowledge or awareness of menarche that girls have about menarche could provide helpful information to educators or health providers for puberty education in the public sector.

## 2 Methods

This hard-copy survey study has been approved by the Ethical Committee of Obstetrics and Gynaecology Hospital, Fudan University of China (reference number 2021-135). The purpose of this survey was thoroughly explained to the participants before they began answering the questionnaire. Additionally, the cover page included an information letter and a statement that participants filled out and returned with the survey, indicating their informed consent. Consequently, the ethical committee waived the requirement for a separate consent form collection due to the nature of the survey study.

### 2.1 Study cohort

A total number of 2,032 adolescent girls (aged from 10 to 17 years) who are living in Shanghai (n = 1,049) and Xiangyun County, Dali City, Yunnan Province (n = 983) participated in this hard-copy survey from October 2020 to May 2021. Shanghai is a relatively wealthy region, while Xiangyun County, Dali city of Yunnan Province, is a relatively low-income region in China. Using a stratified cluster random sampling strategy, six schools in Shanghai and five schools in Xiangyun County of Dali City, Yunnan Province, were selected to participate in this study. This hard-copy survey was completed at an individual school. All participants were informed of the nature and purpose of the study. After a brief instruction on how to complete this questionnaire by the instructors, the girls independently and voluntarily completed the questionnaire in the school classrooms. The questionnaire was designed by epidemiologists and clinicians who are working in the field of obstetrics and gynaecology. There is no personal identification seen in the questionnaire. The absent girls did not participate in this survey at the time of the survey.

The questionnaire included age at data collection, weight, height, the age at the first onset of menstruation, the education levels of both father and mother and the number of siblings. Additionally, the questionnaire included (1) when and where the knowledge of menarche was received; (2) who has been asked for advice if there are any questions about menarche; and (3) how the girls understand the medical conditions of menarche. The detailed questionnaire is in the supplementary file.

Body mass index (BMI) was calculated as the ratio of weight and height (kg/m^2^) at the time of completion of the questionnaire. Most of the menstrual cycle for adolescents is within the range of 21–45 days [6]. In this study, therefore, we defined a longer or shorter menstrual cycle as longer than 45 days or shorter than 21 days.

### 2.2 Statistical analysis

Data on age at the survey, age at the menarche, or BMI was expressed as median/range or mean and standard deviation (SD). The statistical analysis on BMI between the girls with and without menstruation was assessed by *t*-test by GraphPad Prism (version 10.1). P < 0.05 is considered as a statistical difference.

## 3 Results

The demographics of the study cohort are summarised in Table 1. The median age of the girls at the time of the survey was 13 years, ranging from 10 to 17 years. The mean BMI was 18.5 ± 2.7 kg/m^2^. Of these girls, 1,452 (71.5 %) have had menstruation. The median age at menarche for these girls was 13 years, ranging from 12 to 14 years, and the mean age at menarche was 12.7 years. The mean BMI in girls with menarche was significantly greater than that in girls without menarche (19.04 ± 2.58 vs 17.15 ± 2.9 kg/m^2^, p < 0.001).

**Table 1:** The general characteristics of study cohort.

Age at survey (years, median + range)	13 (10–17)
Living conditions (number, %)^a^	
Living with parents	1785 (88.3 %)
Living in a single parent family	103 (5.1 %)
Living with grandparents	134 (6.6 %)
Siblings (number, %)^b^	
One child	883 (43.7 %)
With siblings	1134 (56.3 %)
BMI (mean/SD, kg/m^2^)	18.5 ± 2.7

a data collected from 2,022 answers.

b data collected from 2,017 answers.

We first analyzed when girls received knowledge of menarche. From 1,401 answers, 902 (64 %) girls reported that they acquired knowledge of menarche before their first onset of menstruation. While 499 (36 %) girls did not receive any knowledge of menarche before their first onset of menstruation. Regarding knowledge of the medical definition of menstruation, from 1,431 answers, 401 (28 %) and 907 (63 %) girls fully and partially understood the medical definition of menstruation. In contrast, 123 (8.6 %) girls did not know the medical definition of menstruation. From the survey, we found that 88.7 % of girls received knowledge of menarche from their mothers, followed by friends or classmates (18 %). Interestingly, only 11 % of girls received knowledge of menarche from the school textbooks (Table 2).

**Table 2:** The sources of receiving knowledge about menstruation (multiple options).

	Number (%)
**Mother**	**1,274 (88.7 %)**
Classmates or friends	263 (18.3 %)
Textbooks	157 (11 %)
Television	115 (8 %)
Websites	116 (8.1 %)
Women's magazine	26 (1.8 %)

∗ data collected from 1,436 answers.

We next asked whom the girls would seek information and advice about menarche if they had questions. From 1,442 answers with multiple options, we found that 1,201 (83 %) girls would like to ask advice from their mothers, followed by classmates (20 %) (Table 3). Regarding the emotional impacts of menstruation on girls, from 1,452 answers with multiple options, we found that depression (6.5 %), stress (28.3 %), fear, and anxiety (27.4 %) are the main emotional impacts of menstruation on girls. However, 618 (42.6 %) girls did not have any uncomfortable feelings (Table 4).

**Table 3:** Who to ask about menarche.

	Number (%)
**Mothers**	**1,201 (83.3 %)**
Classmates	283 (19.6 %)
siblings	250 (17.3 %)
Grandparents	64 (4.4 %)
Fathers	14 (1 %)
others	65 (4.5 %)

∗ data collected from 1,442 answers.

**Table 4:** Detailed emotions on menarche and/or menstruation (multiple options).

	Number (%)
No negative feelings	618 (42.6 %)
Easy to be angry	540 (37.2 %)
Exhausted	411 (28.3 %)
Weak	314 (21.6 %)
Muscle soreness	314 (21.6 %)
Anxiety	308 (21.2 %)
Less appetite	232 (15.9 %)
Depression	95 (6.5 %)
Fear	91 (6.2 %)

∗data collected from 1,452 answers.

In this survey with multiple options, we found that 18 % of girls have either oligomenorrhea (longer than 45 days of the menstrual cycle) or polymenorrhea (shorter than 21 days of the menstrual cycle), 21 % of girls have different degrees of dysmenorrhea, and 17 % or 6 % of girls have abnormal menses. In contrast, only 32 % of girls have regular menstruation (Table 5).

**Table 5:** Details of abnormal menstruation (multiple options).

	Number (%)
Irregular menstrual cycle	
oligomenorrhea (longer than 42 days)	147 (10.2 %)
polymenorrhea (shorted than 21 days)	104 (7.2 %)
Menses	
Longer menses (longer than 7 days)	247 (17 %)
Shorter menses (shorted than 3 days)	84 (5.8 %)
Menstrual bleeding heavy	183 (12.6 %)
Dysmenorrhea	302 (20.8 %)
Normal menstrual cycle	468 (32.3 %)

∗data collected from 1,448 answers.

Studies have suggested that economic background plays a significant role in menstruation [12]. We compared the number of girls who had begun menstruating and their age at menarche between those living in Shanghai, a wealthy city, and those in Xiangyun County, Yunnan Province, a relatively low-income region (Table 6). In Shanghai, 630 girls (60 %) had begun menstruating, which was significantly lower than the 83 % in Xiangyun County (p < 0.001). The mean age at menarche for girls in Shanghai was 11.4 years, while in Xiangyun County, it was significantly higher at 11.9 years (p < 0.0001).

**Table 6:** Comparison of menarche associated information between Shanghai and Xiangyun County (Yunan Province).

	Shanghai (n = 1098)	Xiangyun County (n = 983)	
Age at menarche (years, mean/SD)	11.4 ± 0.7	11.9 ± 0.7	p < 0.0001
Beginning menstrual cycle (n, %)	630 (60 %)	810 (83 %)	p < 0.0001
Receiving knowledge about menarche before onset (n, %)	843 (76.8 %)	539 (54.8 %)	p < 0.0001

This survey also found that 53 % of their parents (fathers or mothers) have a tertiary education degree.

## 4 Discussion

In this self-reported hard-copy survey, we found that 72 % of adolescent girls have had menarche, with a median age at menarche of 13 years. Of them, 64 % received knowledge of menarche before their first onset of menstruation, predominantly from their mothers. Mothers were also the primary persons for girls seeking advice and knowledge on menarche. 43 % of adolescent girls had no negative emotions about the menarche. In contrast, the remaining girls reported varying degrees of emotions, such as unhappiness and physical attribute-associated emotions during menstruation. Furthermore, 18 % of adolescent girls had an irregular menstrual cycle.

The current mean age at menarche in well-nourished populations in developed countries is between 12 and 13 years [13,14]. Our recent study reported that the mean age at menarche was 12.7 years, and none of the girls who have not had menarche were over 15 years, consistent with other Chinese studies [10,11] and Western studies [13,14]. Higher weight gain or increased BMI during childhood is related to an earlier onset of puberty [15,16]. Although nutrition data was not collected from this survey, we found that BMI in adolescent girls who have had menarche was significantly higher than that in girls who have not had menarche. In addition, American National Health Statistics Reports recently found that girls from higher socioeconomic families (measured by their mother's educational background) were less likely to reach menarche at an early age than girls from lower socioeconomic families [17]. Interestingly, this finding also applies to the Chinese population. Our current study found that the proportion of fathers with tertiary education was similar to that of mothers. The proportion of fathers or mothers with tertiary education background was significantly lower in girls who have had menarche at the time of the survey, compared to the proportion of fathers or mothers with tertiary education in girls who have not had menarche (44 % vs 74 %).

It is well-recognized that girls should be informed of the body changes, and parents should talk and discuss menarche when the girls reach puberty or before the onset of menstruation. Puberty education, which includes human body development, is widespread in Western countries. These courses provide helpful and valuable knowledge and skills to help girls handle puberty's physical, emotional, and interpersonal changes with positive outcomes (reviewed in Ref. [18]). However, due to cultural barriers, the education program for puberty in China needs to be better formed. This also includes the variety in the program's content and quality [19]. This could result in a larger proportion of girls receiving some knowledge of menarche before the onset of menstruation. In our current study, we found that 36 % of girls aged approximately 11.7 years had yet to receive any knowledge on menarche before their first onset of menstruation. This is also reflected in the understanding of medical conditions of menstruation. By adequately explaining menarche by the family and society, girls may not have a negative experience of menarche [5]. Similar to another Chinese study [20], we found that more than half of girls had different negative emotions about menstruation. This could result in 36 % of girls needing to gain knowledge on menarche. A previous study showed that 75 % of adolescent girls needed to adequately receive knowledge on menarche in China in 2003 [20]. Compared to that study, our current data showed that 64 % of girls acquired knowledge of menarche, suggesting an improvement in puberty education in China.

While parents in the United States of America are actively involved in puberty education for their children [21], a significant percentage of public school teachers (72 %–90 %) undertake the puberty education program [22], suggesting that school can be a source for girls receiving knowledge on menarche. However, this is not the case in the low-and middle-income countries [7]. In our survey, we found a lower proportion of adolescent girls (11 %) receiving knowledge on menarche from school textbooks and teachers. Like studies performed in low- and middle-income countries (reviewed in Ref. [7]), we found that 89 % of adolescent girls receive knowledge on menarche from their mothers. This finding highlights the need for educators and healthcare providers in the puberty education sector in low- and middle-income countries. School teachers and nurses should play an essential role in providing crucial knowledge and skills on menarche to help girls.

Considering the difference in modernization and economic status between Shanghai and Yunan Provinces in China, we compared the proportion of girls who had begun menstruating and their age at menarche between the two sites. We found that the proportion of girls who had begun menstruating in Shanghai was significantly lower than that in Xiangyun County. The age of menarche in girls who live in Shanghai was younger than girls who live in Xiangyun County. This finding indicates that socioeconomic status is associated with menstruation [12]. However, the age of menarche seen in our current study did not practically differ between the two regions. Additionally, we also compared how girls received knowledge about menarche between the two sites. We found there was a significantly higher proportion f adolescent girls who are living in Shanghai receiving knowledge about menarche before their first onset of menstruation, compared to adolescent girls who live in the rural regions (76.8 % vs. 54.8 %). This finding further suggests that puberty education is urgently required in rural areas to promote equity.

The average age of pubertal onset (starts secondary sex characteristics) is around 8 and 9 years for girls [23]. In our current puberty education program, the majority of adolescent girls (82 %) were over 12 years old, and the survey found that 36 % of adolescent girls with a mean age of 11.7 years had not received any knowledge about menarche before their first onset of menstruation. Collectively, this prompts us to question whether our current puberty education program may be initiated too late to be fully effective, especially for adolescent girls who mature early relative to their peers.

Current knowledge suggests that menses is a critical measure of health in adolescent girls, especially within the first three years after onset [24]. This is crucial due to the common occurrence of irregular menstrual cycles (longer than 45 days or shorter than 21 days in the first three years [25]). In our survey, we only found 18 % of adolescent girls having irregular menstruation and 23 % of adolescent girls having abnormal menses. This variation could be due to ethnicity-specific and/or differences in the age of first menstruation between Chinese girls and other ethnicities. In this survey, we also found that 21 % of adolescent girls have dysmenorrhea. The prevalence of dysmenorrhea is not well documented due to difficulties in its definition, but it is estimated range from 45 % to 95 % in adolescent girls [26]. A recent systemic review also reported 17 %–80 % of dysmenorrhea [27].

Several limitations should be taken into consideration when interpreting our findings. Data, including conditions of menstruation and the body weight presented in this study, was self-reported, which could cause a bias. Additionally, we chose not to analyze parents' education level influence on adolescent girls' knowledge of menarche in this study because the regional differences in education and social conditions are well-documented and expected to align with existing findings. However, we acknowledge the value of such an analysis. Our descriptive results did not consider other factors that may cause a difference in our conclusions, such as family economic status and nutritional regime. In addition, given the relatively moderate sample size and the limitation of data collection to only two ethnic groups, our findings may not fully represent China as a whole. With over 56 ethnic groups, China exhibits significant cultural diversity, including variations in nutritional habits. A future multi-center study with a large sample size must validate our findings.

In conclusion, our hard-copy survey study reported that the mean age of Chinese adolescent girls at menarche was 12.7 years. 36 % of girls did not receive any knowledge of menarche before their first onset. In addition, mothers, rather than school teachers and school textbooks, were the primary sources of receiving knowledge and advice on menarche. Our study provides updated information on puberty education showing that it needs to be a high priority in managing adolescent girls’ health, at least at the community level in low- and middle-income countries.

## CRediT authorship contribution statement

**Yifei Gao:** Formal analysis, Data curation. **Jue Wang:** Formal analysis. **Mulan He:** Data curation. **Ruhe Jiang:** Data curation. **Yan Du:** Writing – original draft, Conceptualization. **Qi Chen:** Writing – review & editing, Writing – original draft, Supervision, Investigation, Conceptualization.

## Consent to participate

The ethical committee waived the consent form from participants due to the nature of the survey study.

## Consent to publish

Not applicable.

## Ethical approval

This study has been approved by the Ethical Committee of Obstetrics and Gynaecology Hospital, Fudan University of China (reference number 2021-135).

## Data availability statement section

Data included in this study will be available upon request.

## Funding

None of the authors received any funding for this study.

## Declaration of competing interest

The authors declare that they have no known competing financial interests or personal relationships that could have appeared to influence the work reported in this paper.
