# Supplementary material for: Menarche education and emotional preparedness: A cross-sectional survey study of Chinese adolescent girls
Source: Heliyon. 2025 Feb 21;11(4):e42904. doi: 10.1016/j.heliyon.2025.e42904 (PMC11894302; doi:10.1016/j.heliyon.2025.e42904)
Supplement: Multimedia component 1 [file mmc1.doc]

**Questionnaire for adolescent girls**

1. Age ________ year
2. Height _________ cm
3. Weight _________ kg
4. Place of birth________
5. Living condition

a) □ with parents

b) □ with mother or father

c) □ with grandparents

1. Do you have any siblings?

a) □ Single child

b) □Have one sibling

c) □Have two siblings

d) □Have three or more siblings

1. Mother’s highest education：

a)□ Elementary school or less

b)□ Junior high school/technical school/vocational school

c)□ Senior high school

d)□ Junior college

e)□ College

f)□ Graduate school

1. Father’s highest education：

a)□ Elementary school or less

b)□ Junior high school/technical school/vocational school

c)□ Senior high school

d)□ Junior college

e)□ College

f)□ Graduate school

1. Mother’s occupation：

a)□ Worker or service industry employee

b)□ Private enterprise or small business owner

c)□ Professional or administrative staff

d)□ Unemployed

e)□ Other________

1. Father’s occupation：

a)□ Worker or service industry employee

b)□ Private enterprise or small business owner

c)□ Professional or administrative staff

d)□ Unemployed

e)□ Other________

1. Have you had menarche?

a) □Yes，age at menarche ____year（Please continue answering the following questions.）

b) □No (End of the survey)

1. Knowledge about menstruation:

a)□ Acquired relevant knowledge before menarche

b)□ Acquired relevant knowledge after menarche

1. Source of menstruation knowledge:

a)□ Mother (or other family members)

b)□ Classmate(s) or friend(s)

c)□ School (including textbooks, teachers, health providers, and school programs)

d)□ Women’s magazine

e)□ Television

f)□ Website

1. Do you understand how is the menstrual process formed?

a)□ Fully understand

b)□ Partially understand

c)□ Do not understand

1. Place of menarche:

a)□ School

b)□ Home

c)□ Public place

1. How do you feel about menstruation? (multiple choice)

a)□ Muscle soreness

b)□ Less appetite

c)□ Easy to get angry

d)□ Anxiety

e)□ Fear

f)□ Depression

g)□ Weak

h)□ Exhausted

i)□No negative impacts

1. Do you have any medical conditions about menstruation? (multiple choice)

a)□ Irregular menstrual cycle

b)□ Oligomenorrhea (＞45 days)

c)□ Polymenorrhea (＜21 days)

d)□ Longer menses (＞7 days)

e)□ Shorter menses (＜3天)

f)□ Dysmenorrhea

g)□ Heavy menstrual bleeding

h)□ Normal menstrual cycle

1. To whom willyou seek advice on menarche and/or menstruation?

a)□ Mother

b)□ Father

c)□ Grandmother

d)□ Sister(s)

e)□ Classmate(s)

f)□ Other________
